# Advances in Human Placental Biomechanics

**DOI:** 10.1016/j.csbj.2018.08.001

**Published:** 2018-08-24

**Authors:** R. Plitman Mayo

**Affiliations:** a Homerton College, University of Cambridge, Cambridge CB2 8PH, UK; b Centre for Trophoblast Research (CTR), Department of Physiology, Development and Neuroscience, University of Cambridge, Cambridge CB2 3EG, UK

**Keywords:** Placenta, Biomechanics, Blood flow, Nutrient transport, 3D imaging, Computational modelling

## Abstract

Pregnancy complications are a major clinical concern due to the related maternal and fetal morbidity. Many are caused through defective placentation, but research into placental function is difficult, principally because of the ethical limitations associated with the *in-vivo* organ and the difficulty of extrapolating animal models. Perfused by two separate circulations, the maternal and fetal bloodstreams, the placenta has a unique structure and performs multiple complex functions. Three-dimensional imaging and computational modelling are becoming popular tools to investigate the morphology and physiology of this organ. These techniques bear the potential for better understanding the aetiology and development of placental pathologies, however, their full potential is yet to be exploited. This review aims to summarize the recent insights into placental structure and function by employing these novel techniques.

## 1 Introduction

The placenta is a complex organ that performs a critical function: nourishing a developing baby. It attaches to the uterine wall and connects with the foetus *via* the umbilical cord, growing and adapting during pregnancy to meet fetal demands. Apart from providing the foetus with oxygen, the placenta performs many other vital tasks: for example, it shields the foetus from maternal immune attack *in utero* and fulfils excretory, endocrine, catabolic and absorptive functions [1]. The placenta is the organ that displays most interspecies variation, with the different types sharing only one essential feature: the existence of two separate circulatory systems, the maternal and fetal placental circulations [1].

Studying placental function is far from just an academic exercise. Placental complications can have fatal outcomes for both mother and baby. In fact, 50% of the ~3000 stillbirths in the UK each year result from pregnancy disorders and conditions affecting the placenta [2]. A deficient placental function can result in poor fetal growth which accounts for at least one-third of perinatal deaths in the UK (~1500*per annum*). A further 40,000 pregnancies are complicated by fetal growth restriction (FGR) or pre-eclampsia (PE) [1].

Three factors considerably complicate research into the functional morphology and physiology of the human placenta. Firstly, *invivo*research is strongly restricted by ethical constraints, but also by the limits of resolution of the ultrasound scan which is the only imaging test routinely performed during pregnancy. Secondly, *exvivo* research depends on the availability and accessibility of and to the organ, which in turn depends on hospital protocols, number of volunteers, patient history and mode of delivery. Additionally, an *exvivo* placenta needs to be manipulated as soon as it is collected to avoid the collapse of its vascular structure [3]. Lastly, due to species differences in body size, duration of pregnancy, litter size and living conditions, the shape, structure and biochemistry of the placenta differs considerably even among otherwise closely related species [1], limiting the validity of animal models. As a consequence, there is a pressing need for novel and powerful investigative techniques that can uncover the aetiology of pregnancy complications. This article, therefore, summarises the latest developments and knowledge gained through the combination of two emerging technologies that have improved our understanding of placental morphology and function: 3D imaging and computational modelling.

## 2 Human Placental Morphology

Key to our understanding of the source of pregnancy complications is the full comprehension of placental structure-function relationship in healthy pregnancies.

A typical full-term delivered placenta is a round to oval, flat organ. Its average measurements are 513 g in disc weight [4], 22 cm in diameter, 2.5 cm in thickness at the centre, and has a surface area of almost 15 m^2^ [5]. However, there are significant inter-individual variations in these measurements [1]. The placenta has two surfaces, the chorionic plate that faces the baby and to which the umbilical cord is attached, and the basal plate that is apposed to the uterine wall.

Placental shape has been regarded as round or elliptical but functionally unimportant [6,7]. Nevertheless, increased variability of placental shape has been associated with lower placental efficiency, ahypothesis supported by either uteroplacental or feto-placental vascular pathology [6]. A recent study of 2120 women found a relationship between placental surface area and weight, with uterine and umbilical blood flows, both of which are associated with fetal growth rate [8]. Another study of 916 women found correlations between the surface area of the chorionic plate and its perimeter with birth weight [9], suggesting that these comparatively simple measurements can identify suboptimal placental development. Although umbilical cord insertion is assumed to happen at the centre of the chorionic surface, a recent study found that the cord is actually not centred [7]. However, no relation to adverse outcomes was found [7,10].

The chorionic arteries and vein branch from the umbilical cord towards the basal plate creating about 65 villous stems, each then branching into multiple intermediate villi [1]. The villous trees are regarded as the main functional units of the placenta since they represent the principal site of maternal-fetal exchange [1]. Placental villi are commonly differentiated by their calibre, architecture, position and function. However, despite their classification, all villi exhibit the same basic feature: a villous membrane that separates the maternal and the fetal circulations [1]. The villi undergo differentiation throughout gestation resulting not only in different villous types but also in a huge rise of the villous surface area and thinning of the membrane [11,12]. The linear growth of the terminal villi, which begins in the second trimester, results in a reduction of the maternal-fetal barrier to about 4 μm at the vasculosyncytial membrane near term [12].

Due to its functional importance, understanding the role of a terminal villus geometry is crucial to better understand the efficiency of the placenta as an organ of exchange. Many critical aspects of placental transport are unknown: the interconnectivity of the fetal capillary network, the speed and directions of the two involved flows (maternal and fetal), the extent of the feto-capillary structural variability within a placenta and between different individuals, among others. These aspects are fundamental for efficient transport as they enhance diffusional exchange and therefore, abnormalities in the villous tree structure are associated with placental pathologies.

### 2.1 Three-dimensional Imaging of the Villous Trees

Three-dimensional imaging modalities are emerging as reliable tools to better asses the architecture of the feto-placental vasculature. In fact, by reconstructing light microscopy images, Haeussner etal. [13] demonstrated that placental histology is susceptible to significant inter-individual observations and inaccuracies. Subsequently, different 3D approaches have been proposed to characterise placental villous trees.

In order to investigate the global feto-placental vasculature, micro-computed tomography (μCT) [14] and magnetic resonance angiography (MRA) [15] have been proposed as potential and convenient tools (seeFig. 1d & e, respectively). After evaluating different contrast agents, optimising the imaging protocol and ensuring repeatability, Chen etal. [15] proposed pump oil as an economical and efficient contrast agent for MRA. These techniques allows the visualisation, reconstruction and quantification of up to 6 generations of placental villi. However, if the purpose is to investigate the smaller branches of the villous tree, a more suitable approach would be to use microscopic imaging techniques.

**Fig.1:**
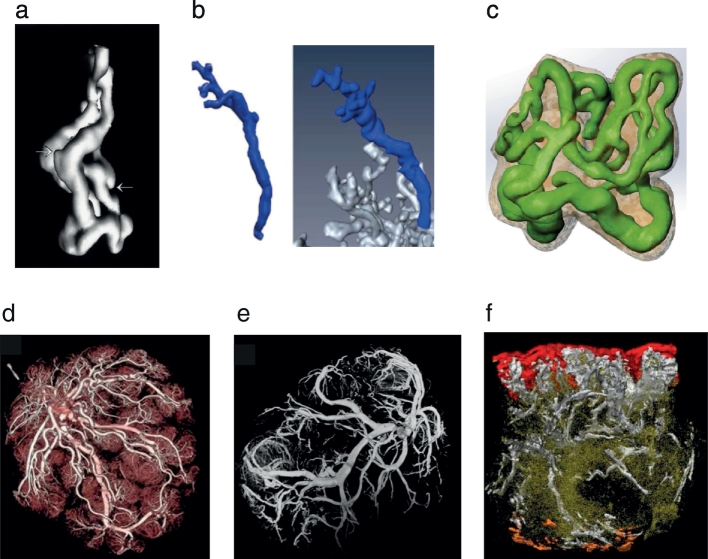
Three-dimensional reconstructions of the human placenta using different imaging techniques. (a)Vasculature of a single terminal villi from CLSM, highlighting capillary buds [16]. (b)3D visualisation of a villous tree from light microscopy images [13]. (c)The complex vasculature of a terminal villi reconstructed from fluorescent CLSM and used for computational simulations [19]. (d)Reconstruction of the global feto-placental network from CT angiography [23]. (e)3D rendering from MRA [15]. (f)Reconstruction of a whole materno-fetal exchange unit from histological sections: chorionic plate (red), basal plate (orange), stem villi (white) and lower order villi (green) [24]. All figures were reproduced with permission.

The pioneering works of Jirkovská etal. [16,17] reconstructed thethree-dimensional structure of terminal villi using confocal laser scanning microscopy (CLSM) images (see Fig. 1a). The different feto-capillary loops and their spatial arrangement in normal placentae were revealed, highlighting the potential of this technique; however, no structural analysis was performed. In order to quantify the villous tree geometry, reconstructions from light microscopy were proposed as an efficient tool [18]. Parameters such as branching hierarchy, branching angles, diameters and lengths of the terminal villi can be obtained using this technique (see Fig. 1b) [18]. Plitman Mayo etal. [19] used fluorescent CLSM images to reconstruct the 3D architecture of the feto-capillary network and the villous membrane in terminal villi (see Fig. 1c). Averaged capillary to villi volume and area fractions were found to decrease with the fixation pressure of the fetal circulatory system, in agreement with previous studies [20]. Following the success of fluorescent CLSM, Merz etal. [21] proposed to clarify the tissue prior to imaging to allow a deeper field of view. High-quality images of several villi were obtained by combining the Visikol® HISTO^TM^technique [22] with fluorescent CLSM in healthy samples from 8, 12, 18, 22 weeks and term placentae.

Three-dimensional imaging has also been used to investigate structural differences in pathological placentae. Using CLSM Jirkovská etal. [25] examined the topological and spatial differences between normal and diabetic placentae (gestational diabetes). Although the results were qualitative, more complicated branching patterns and a large number of redundant connections were found in the pathological samples. A subsequent work by the same authors [26] looked at placentae from type 1 diabetes mellitus, and reported that these show amplification of surface area by enlarging the capillary diameters and creating higher branching.

Placentae from FGR have attracted much attention due to their marked difference in size and structure when compared to normal placentae. Three-dimensional light microscopy images showed that the branching angle and tortuosity of the terminal villi was significantly different between normal and FGR samples [18]. Micro-CT has also been tested as an effective technique to analyse the vascular structure of pathological placentae [27]. Significant differences were found between normal and FGR samples. Mainly, the vascular volume fraction was lower and of a similar magnitude in the different areas of the placenta [27]. In an effort to improve imaging quality and resolution, Junaid etal. [28] imaged casts of the feto-placental vasculature with a micro-CT machine finding that arteries and veins were shorter and longer, respectively, in FGR than in normal placentae. However, if the contrast between the tissue and its surrounding is not strong enough, three- dimensional reconstructions can become a tedious and lengthy process. To allow an automatic reconstruction of placental images, Thunbo etal. [23] injected a contrasting mixture to the feto-placental vasculature and imaged the villous trees using computed tomography angiography (CTA). In this work, FGR placentae showed an increase in macrovascular density, calculated as the ratio of macrovascular parameters (vascular surface area and the number of vessel junctions) and placental volume.

An entirely different approach was taken by McCarthy etal. [24] who tested the capabilities of 3D reconstruction from histological sections(seeFig. 1f). Using a customised software, the authors reconstructed placental samples from healthy and complicated pregnancies (*n* = 1each for pre-eclampsia, gestational diabetes and FGR). Although marked differences were noted between the samples, a detailed quantitative morphological analysis was out of the scope of the work.

The complexity of placental vasculature and its change during disease has intrigued researchers for a long time. There are differences in morphology associated with complications but quantifying them is difficult due to the complex 3D structure, and understanding exactly how these differences are related to pathologies is challenging and needs detailed experimental work. Additionally, little is known on how these changes impact placental haemodynamics.

## 3 Maternal and Fetal Blood Flows

The placenta displays unique haemodynamic characteristics. Firstly, it has two blood supplies, maternal and fetal, and mass exchange is accomplished by diffusion across thin membranes separating the two. The fetal blood circulates through a network of capillaries contained within the placental villous trees. Each tree is centred over a maternal arterial opening, forming a series of 30–40 individual maternal-fetalexchange units [1]. Second, the maternal blood is released into a cavity surrounded by villi, commonly known as the intervillous space. It percolates between the villi before draining into the decidual veins.

The maternal and fetal placental circulations develop independently, and a complete feto-placental circulation is only established around the beginning of the sixth week post-conception (p.c.). However, effective flow and exchange is believed to only start at the end of the first trimester [1]. The direction, distribution and patterns of the maternal and fetal flows have a major impact on the capacity of the placenta as an organ of exchange. In fact, the fast diffusion of respiratory gases through the trophoblastic membrane implies that it is the maternal and fetal flows that are responsible for maintaining a crucial diffusion gradient. Therefore, the human placenta has been described as flow-limited rather than diffusion-limited [29,30].

Significant efforts have been put into better understanding theplacental circulations and their implications for the functionality of the organ. The following sections review how, in recent years, computational modelling has improved our knowledge of placental blood flows.

### 3.1 Maternal Placental Circulation

The maternal placental circulation has been described as the source of some pregnancy complications [1], and therefore, understanding its haemodynamics has been of much interest. Vascular casting demonstrated that maternal blood is delivered from the spiral arteries (SA) into the villus-free central cavity of each maternal-fetal exchange unit, where it then disperses radially between the villi [31]. Maternal blood flow paths are affected by the position of the villi, leading to a decrease in flow rate from the basal to the chorionic plate and from the centre towards the periphery [1]. The intervillous space (IVS) has no specially designed channels for blood to flow in, rather it is acknowledged as a 'pool' of blood where blood flow is driven by local haemodynamic pressure gradients [32]. Therefore, the direction of the stream at any given point in the IVS is determined by the locations of the arterial and venous openings, and the geometry of the maternal-fetal exchange unit [33].

Due to the inaccessibility to the living organ and the complications of reproducing placental perfusions *ex-vivo*, mathematical and computational models have been very popular to investigate maternal and fetal placental circulations. In the early days, placental circulations were modelled as compartmental models with five principal flow patterns [[34], [35], [36], [37], [38], [39], [40]]: pool flow, double pool flow, countercurrent flow, concurrent flow and cross-current flow. These previous attempts have simplified the system greatly, some so much that the model bears little physiological significance to the biology. However, recent advances in imaging techniques and computational power have led to much more sophisticated models that can be split into two main groups: models looking at the blood movement adjacent to a spiral artery opening - or intervillous flow - and models focusing on micro-circulation involving single villi branches.

**Fig.2:**
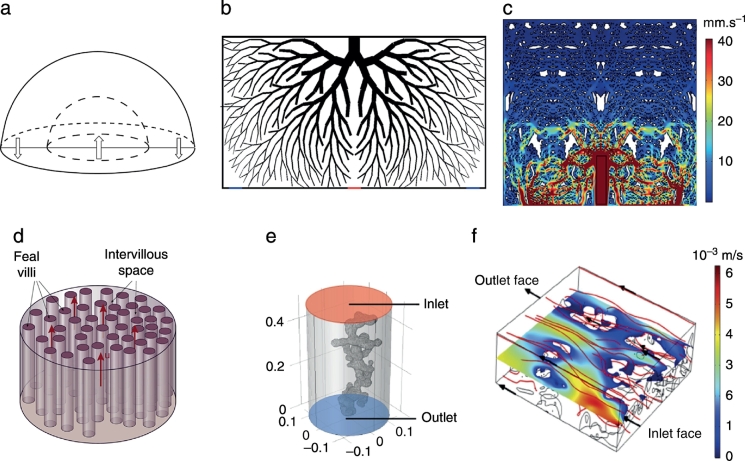
Models of maternal placental blood flow. (a)3D hemispherical model to simulate flow in the IVS [42]. (b)Two-dimensional parametric model of a villous tree with a SA opening in the middle (red) and two draining veins at the sides (blue) [45]. (c)Histology based model of the IVS blood flow [46]. (d)Stream-tube model where terminal villi are represented by small parallel tubes [47]. (e)Single terminal villi inside a cylinder of maternal blood [46]. Three-dimensional CLSM-based model of blood flow through several villi [48]. All figures were reproduced with permission.

Following the pioneering work from Erian etal. [41], most of the IVS models are 2D and include a spiral artery opening to a field of porous tissue with a central cavity and draining veins located at the sides of the artery. However, two-dimensional models oversimplify the complexity of the IVS and so, Chernyavsky etal. [42] created a three-dimensional hemispherical domain to look at the flow and solute concentrations (see Fig. 2a). This work shows that the central cavity helps to alleviate high stresses in the immediate neighbourhood of the decidual artery and veins, and that the calibre of the spiral artery and decidual veins control the pressure difference in the surroundings of the opening. This model was later reproduced to better understand the relationship between blood jets and villous density by changing the porosity of the tissue – simulating changes during gestation [43]. They concluded that by the end of gestation, when the flow becomes stronger, there is a decrease of tissue density around the spiral artery and as a consequence, it is reasonable to assume that maternal blood flow shapes villous tree development. This outcome is in agreement with the view of Reynolds etal. [44] who reported that lobules- materno-fetal exchange units defined by the boundaries of septa- are not seen until the start of the 2nd trimester when arterial inflow commences.

To investigate the interaction between IVS blood flow and the villous tree, more sophisticated models that explicitly include the structure are needed. For this purpose, Lin etal. [45] created a two-dimensional model, that resembles the villous tree architecture, using an in-housealgorithm. The structure included intermediate villi while terminal villi were modelled as homogenised blocks (see Fig. 2b). This model demonstrated that a branch angle of 24° is optimum for nutrient exchange since it provides a balance between maximising surface area and allowing sufficient blood flow penetration. Other models have looked into the wall shear stress distribution on the villous surface. For this purpose Lecarpentier etal. [46] created a three-dimensional model similar to that of Chernyavsky etal. [42] but including viscous effects. Additionally, they created a 2D model based on a typical histological section, providing the first IVS model with a realistic structure (see Fig. 2c). These models predicted that a low WSS is applied to the villous surface despite the high maternal blood flow rates and revealed various flow patterns.

Three-dimensionalimage-based models of IVS have also been used to investigate the effect of impaired SA remodelling on maternal blood flow [49]. Using velocity waveforms from Doppler data, IVS blood flow was calculated and compared between normal and FGR cases. Turbulent flow with high-velocity jets and vortices, developing due to the insufficient remodelling of SA, were found in the IVS for the pathological cases. This might explain the marked differences in placental structure for FGR, which grow according to an altered maternal flow.

At the single villous scale (≥300 *μ*m), maternal blood is assumed to be moving relatively slow to allow for exchange processes. However, the intervillous space is a highly heterogeneous structure and when looking at a single or only a few villi, homogenization approximations introduce inherent errors. To better understand the implications of these, Chernyavsky etal. [50] created a one-dimensional (1D) array of point sinks, representing the terminal villi, where maternal blood was assumed as unidirectional flow and solute transport was analysed. A similar approach was later used to examine the optimal villi density with regards to oxygen exchange [47]. The later model consisted of maternal blood flowing through a 3D large cylinder containing multiple smaller parallel tubes representing the terminal villi (see Fig. 2d). An optimal villi density of 0.46 ± 0.06 was shown to provide the best trade-off between maximising the surface area for exchange and sufficient space for maternal blood to percolate.

Realistic terminal villi geometry has been reconstructed from 3D images and converted into models with the aim of analysing nutrient transport mechanisms. Lecarpentier etal. [46] created a 3D model of a single terminal villus floating inside a cylinder of maternal blood (seeFig. 2e) based on scanning electron microscopic images; however, the impact of neighbouring villi and villous density is neglected. A way to overcome this is by reconstructing a block of villi, such as in the work of Perazzolo etal. [48]. Three-dimensional reconstructions of IVS from CLSM images were coupled with computational modelling (seeFig.2f). This study provides a better understanding of the implications of placental structure in the transfer of substances, such as the fact that less dense microvilli had a reduced surface area for exchange but is compensated by increased flow.

Maternal placental flow is the source of nutrients for the developing foetus and therefore, one of the most critical aspects of the organ. Assessing the haemodynamic properties of placental flow *invivo* has been challenging due to ethical constraints and limits of resolution. Computational modelling has become the leading technique for analysing maternal blood movement in the human placenta. However, there is still a gap in our understanding of the relationship between maternal placental flow and oxygen supply to the foetus, the fetal bloodstream and the healthy development of the baby.

### 3.2 Feto-Placental Blood Flow

The feto-placental circulation refers to the bloodstream along theumbilical arteries, moving through the chorionic vasculature onthe way to the terminal villi, and returning to the fetal circulation *via* the umbilical vein.

Umbilical flow models became very popular to explain the high resistance index in pathological pregnancies after Doppler ultrasound was proposed as an early risk assessment tool [51]. Kleiner-Assaf etal. [52] created an elastic cylinder with thin walls to simulate an uncoiled umbilical artery. This model showed a linear relationship between arterial pressure at the placental insertion, blood viscosity, and maximal pressure in the umbilicus with Doppler indices (DI). Additionally, vessel wall modulus, blood density, and mean arterial pressure in the umbilical cord were found to be inversely proportional to DI. A similar coiled model was subsequently proposed by Kaplan etal. [53] to analyse the haemodynamics of arterial blood flow. A relationship between driving gradient pressures and the coils' pitch was found, together with spatial gradients of WSS along the cross-sections. Umbilical cord complications have also been investigated using computational modelling. Athree- dimensional model to study Hyrtl anastomosis - a connection between the umbilical arteries near the cord insertion - was proposed by Gordon etal. [54]. Two straight tubes with transverse connection ending in cylindrical porous media, representing the arterial vasculature, were created. This study concluded that Hyrtl anastomosis redistributes blood flow or pressure to improve placental perfusion.

**Fig.3:**
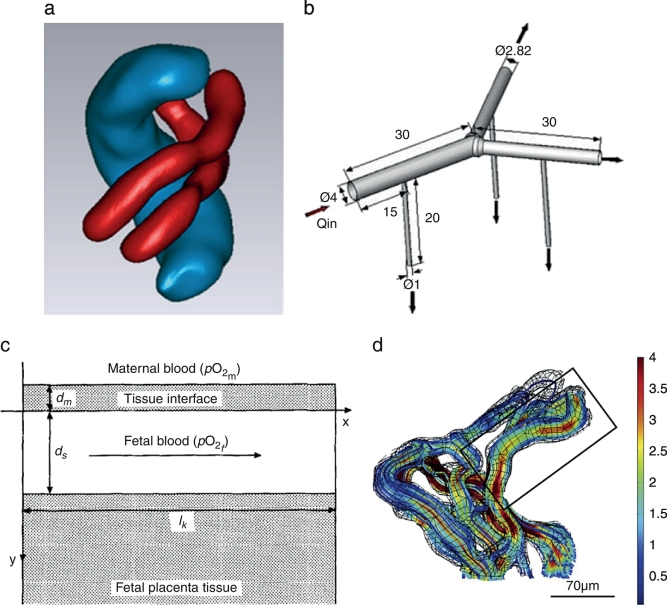
Models of feto-placental blood flow. (a)Ultrasound based model of the umbilical arteries (red) and vein (blue) [55]. (b)Parametric model of a dichotomous branching in the chorionic arteries [57]. (c)One-dimensional model of a placental transport unit [58]. (d)Three-dimensional CLSM based model of a terminal villi with flow streamlines [mm/s] [59]. All figures were reproduced with permission.

The growing capabilities of imaging techniques and the potential of 3D reconstructions have significantly improved modelling of the feto-placental blood flow. A recent work was able to reconstruct the three-dimensional geometry of the umbilical arteries and vein from 3D B-mode ultrasound images (see Fig. 3a) and to couple it with computational simulations [55]. Interestingly, this work found no correlation between umbilical artery WSS and diameter, while it did for the umbilical vein. The authors suggested that the umbilical cord shape might be an adaptation feature to preserve WSS and flow when under mechanical loadings such as in cases of compression and bending.

Little attention has been given to the feto-placental circulation in the villous tree vasculature due to the inaccessibility to structural and experimental data. Using a parametrised multi-scale model of the feto-placental vasculature, Clark etal. [56] showed that the shape of the placenta does not affect placental efficiency. The branching structure was created using an in-house algorithm and included six to eight generations with 60–100 villous trees; terminal villi where taken into account as distal resistances. In an attempt to better understand the physiological significance of the villous tree branching patterns, Gordon etal. [57] created 3D models for the dichotomous(bifurcation branching) and monopodial (daughter tubes branching from the main tube) segments of the chorionic arteries (see Fig. 3b). Monopodial segments were found to be efficient in long distance delivery of blood, while dichotomous branching was more efficient in distributing the blood near the bifurcation.

Fetal blood motion in the terminal villi has barely been explicitly solved and analysed. Therefore, when modelling the feto-placental blood flow, the terminal villi are usually taken as constant resistances. One of the earliest villi models tried to overcome this problem by creating a 3D idealised model of maternal and fetal blood flows [35]. Although the geometry was simplified, the model included both circulations and the interaction between dissolved and bound oxygen in the maternal and fetal streams. The outcome was then coupled with a complete fetal circulatory system. A simpler approach is to model terminal villi blood flow as a 1D fluid [58]. With the aim of understanding the oxygen mechanisms during the second trimester, where data are scarce, Costa etal. [58] took the fetal capillary as a flat-wall exchanger and blood flow as laminar or whirling finding that the human placenta is less sensitive to pathologies at term than in early gestation.

The structure-function relationship of the terminal villi has been recently analysed using 3D reconstructions from CLSM (see Fig. 3d) [59]. These models focused on how the unique structure of the terminal villi vasculature impact the motion of blood and oxygen transport. Sinusoids - a key feature of the terminal villi - are shown to decelerate the blood flow by up to 80% while thinning the barrier and therefore, allow faster and better oxygenation of the fetal blood. Additionally, vortical flow was shown to be physiologically impossible at the terminal branches [59]. A similar approach was taken by Pearce etal. [60] who demonstrated that oxygen transport is maximised by an optimal dilatation of the capillary when the pressure drop remains constant. Additionally, this work calculated the vascular resistance of the terminal villi which can then be used as a boundary condition for multi-scale models, such as the one created by Clark etal. [56].

Computational simulations have been shown to be a convenient tool to investigate different aspects of the feto-placental circulation. The implication of Hyrtl anastomosis [54], the function of the different branching patterns commonly found in the chorionic plate [57] or the role of the unique structure of the terminal villi [59], are just examples of the capabilities of computational modelling. However, the relationship between the feto-placental bloodstream and the capacity of the placenta to efficiently supply the foetus with nutrients is still unclear.

## 4 Placental Transport Function

The primary function of the placenta is to provide the developing baby with nutrients. During several pregnancy complications, the nutrient supply to the foetus is disrupted, however, there is little information on how and why this disturbance takes place. This has motivated researchers to model the transport function of the human placenta. Although there are several transport mechanisms involved in transport through the placenta, this review focuses on passive diffusion of oxygen molecules due to the great attention that it has attracted.

**Fig.4:**
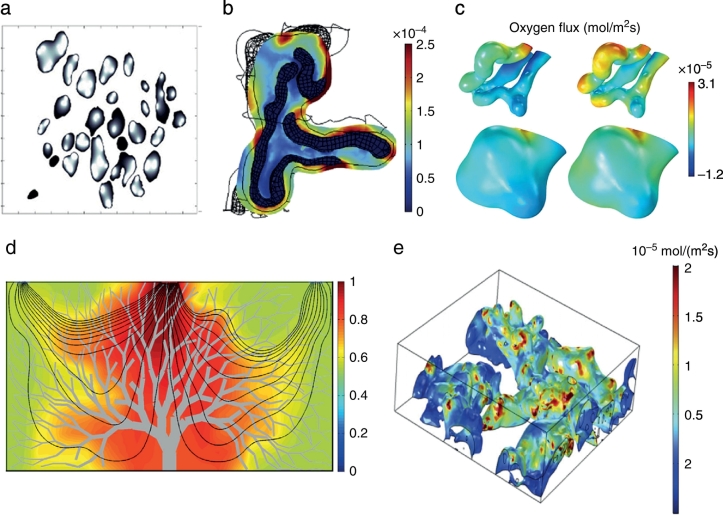
Models of transport in the human placenta. (a)Oxygen concentration map in terminal villi; darker shading represent higher oxygen concentration [61]. (b)Oxygen flux [mol/m^2^*s] across the villous membrane [19]. (c)Oxygen flux across the capillary surface of the feto-vasculature in a terminal villi [60]. (d)Flow streamlines and oxygen concentration map in the intervillous space [45]. (e)Oxygen flux in terminal villi due to maternal blood flow [48]. All figures were reproduced with permission.

The pioneering work of Gill etal. [61], who used a segmented histology slice for simulating oxygen concentrations in the terminal villi (seeFig. 4a), opened the way for a new era of placental transport models. The concept of diffusional screening, whereby some capillaries are ‘shielded’ from receiving oxygen was then introduced. Plitman Mayo etal. [19] simulated a similar scenario but on 3D structures reconstructed from CLSM images (see Fig. 4b), and confirmed the previous findings on diffusional screening [61]. These models were subsequently upgraded by including the fetal blood flowing through the capillary network [59]. The drastic changes in diameter, commonly seen in the terminal villi vasculature, where shown to decelerate the flow and suggested to be an effective way of maximising the time for oxygenation without affecting the oxygen supply to the foetus. Similar models were proposed by Pearce etal. [60] (see Fig. 4c), who quantified the sensitivity of these simulations to uncertainties in the geometry and parameters.

Oxygen transport has also been modelled by calculating the maternal bloodstream and taking the fetal vasculature as perfect sinks. Serov etal. [47] created the stream-tube model (see Fig. 2d) where the structure of the terminal villi was simplified to parallel tubes contained within a big cylinder, representing the intervillous space. This study predicted the best trade-off between adequate maternal blood flow and oxygen transport to be a villi density of 0.47 ± 0.06. In an attempt to provide a more realistic model, Lin etal. [45] created an average villous tree model using published structural data (seeFig.4d). This study predicted a small influence of the villous tree branching generations on the oxygen uptake and a preferential branch angle of 24°. The latest and more sophisticated model, reconstructed a block of CLSM images [48] that included several villi, following the methodology proposed by Plitman Mayo etal. [59]. Solute transport was simulated in the intervillous space with different maternal scenarios while fetal blood flow was neglected (see Fig. 4e). This study suggests that ignoring the maternal blood flow leads to a 2.4 ± 0.4 fold overprediction of transfer and that maternal blood is a limiting factor in transfer across the placenta.

## 5 Outlook

There has been significant progress in our understanding of the structure-function relationship of the human placenta. However, there is still much work required to fully asses the role of the unique arrangement of the placenta in its ability to successfully nourish a growing foetus.

Characterisation of the vascular architecture and determination of its topological principles are essential to improve our understanding of the role of the blood vessels network in pathological conditions. Several aspects of the micro-circulation such as oxygen transport, pressure and wall shear stress distribution, and blood flow regulation highly depend on the vascular network anatomy, and therefore, detailed structural information is required. Once the characterisation of the healthy feto-capillary network is completed, structural comparisons between normal and pathological placentae should be performed. Quantitative data on the placental architecture across conditions can then elucidate placental pathologies aetiology and development.

There is also the need to improve the computational models in order to achieve a more accurate description of the dynamic behaviour and function of the human placenta. Although laminar flow can be assumed in the terminal villi due to the small Reynolds number, the variation in red blood cells concentration and fluid viscosity due to diameter variability have a significant effect on the flow patterns. These phenomena are known as the Fåhræus and the Fåhræus-Lindqvist effects, respectively [62]. Including these in the computational models will yield to an accurate wall shear stress distribution in the vascular network. The implementation of these effects in models with complex geometries is challenging, mostly due to their dependency on capillary diameter. However, a recent study [63] succeeded in modelling these phenomena in a late gestation rat feto-placental arterial network reconstructed from CT images. This novel study found wall shear stress magnitude and gradients at bifurcations, which may contribute through gene activation to vessel enlargement, and sprouting and pruning during angiogenesis. The vascular resistance could then be calculated directly from these physiologically-realistic simulations. Neglected mechanisms that may contribute to the overall oxygen transport capacity and efficiency of the placenta should also be modelled. Placental tissue is known to be a highly metabolic tissue [64,65] which is, therefore, a potentially limiting factor on the oxygen transport to the foetus. In fact, it has been demonstrated that the more oxygen provided to the placenta, the more it consumes [65]. Since only a few experimental methods succeeded to elucidate on the extent of placental oxygen consumption [66], reverse engineering can become a potentially tractable approach to calculate the amount of oxygen that a placental tissue volume unit is absorbing. The double Bohr effect, known to be present only in the placenta [36] is usually neglected for simplicity purposes. However, since the saturation of blood is directly related to the amount of dissolved and bound carbon dioxide (CO_2_) and its unbinding rate from the haemoglobin, it is expected that this effect will limit the transport capacity of the terminal villi. Theoretical models of CO_2_ transfer in the placenta and its interaction with oxygen were created by Hill etal. [36] using a one- dimensional model. Nowadays, computational power and image analysis allow the implementation of Hill's theoretical model in realistic 3D geometries. Computational simulations can also help to understand the mechanisms involved in pathological scenarios, such as in the case of hypoxic vasoconstriction, whereby fetal placental vessels constrict in response to maternal placental hypoxia [67]. The effect of the different oxygen tensions in the IVS on the fetal placental blood flow in such scenarios, is currently unknown.

Additionally, the expansion and contraction of the placenta due to pressure fluctuations needs to be included in future models. The deformation of both the villous membrane and the feto-placental capillary network probably control the motion of the maternal and fetal blood and the vascular resistance. However, the stiffness of placental tissue and its components is currently unknown.

## 6 Conclusions

Novel imaging techniques and computational modelling are becoming popular tools to address questions on the performance of the placenta and its relationship to its unique structure.

Three-dimensional imaging has allowed the quantification of some intriguing geometrical parameters of the terminal villi [18,19]. Additionally, a more in-depth look at the structural differences between healthy and pathological placentae has been possible [14,18,23,26,28].

Computational simulations have broadened our understanding of the structure-function relationship in the human placenta. A villous tree density of 0.46 with a branch angle of 24° was found to be optimal for maximising oxygen transport [47,48]. On the other hand, the umbilical cord shape is suggested to be ideal for preserving WSS when under mechanical loading [55], while the drastic changes in feto-capillary diameters were shown to decelerate the flow allowing blood oxygenation [19]. Some of the underlying mechanisms of placental function have also been explained using computational simulations. The lobule central cavity was shown to relieve high stresses in the surrounding villi [42] and impaired remodelling of SA was suggested to create turbulent flows [49], explaining the lower efficacy of pathological placentae. The overall shape of the placenta is believed to be irrelevant [56], however, the different branching patterns of the fetal placental vasculature do have specific haemodynamic functions [57].

The full potential of image-based modelling in placenta-relatedresearch is yet to be realized.

## Competing Interests

The author declare no conflict of interest.

## Acknowledgements

The author gratefully acknowledges the support Homerton College and the Centre for Trophoblast Research (CTR), University of Cambridge, UK. Additionally, the author would like to acknowledge Prof. Graham Burton and Dr. David Labonte for their helpful suggestions.
